# Effects of Diffuse Light on Radiation Use Efficiency of Two *Anthurium* Cultivars Depend on the Response of Stomatal Conductance to Dynamic Light Intensity

**DOI:** 10.3389/fpls.2016.00056

**Published:** 2016-02-04

**Authors:** Tao Li, Johannes Kromdijk, Ep Heuvelink, F. R. van Noort, Elias Kaiser, Leo F. M. Marcelis

**Affiliations:** ^1^Horticulture and Product Physiology Group, Wageningen University and Research CentreWageningen, Netherlands; ^2^Institute of Environment and Sustainable Development in Agriculture, Chinese Academy of Agriculture SciencesBeijing, China; ^3^Carl R. Woese Institute for Genomic Biology, University of IllinoisUrbana, IL, USA; ^4^Wageningen UR Greenhouse HorticultureWageningen, Netherlands

**Keywords:** diffuse light, temporal light distribution, radiation use efficiency, dynamic leaf photosynthesis, stomatal conductance, *Anthurium andreanum*

## Abstract

The stimulating effect of diffuse light on radiation use efficiency (RUE) of crops is often explained by the more homogeneous spatial light distribution, while rarely considering differences in temporal light distribution at leaf level. This study investigated whether diffuse light effects on crop RUE can be explained by dynamic responses of leaf photosynthesis to temporal changes of photosynthetic photon flux density (PPFD). Two *Anthurium andreanum* cultivars (‘Pink Champion’ and ‘Royal Champion’) were grown in two glasshouses covered by clear (control) and diffuse glass, with similar light transmission. On clear days, diffusing the light resulted in less temporal fluctuations of PPFD. Stomatal conductance (*g*_s_) varied strongly in response to transient PPFD in ‘Royal Champion,’ whereas it remained relatively constant in ‘Pink Champion.’ Instantaneous net leaf photosynthesis (*P*_n_) in both cultivars approached steady state *P*_n_ in diffuse light treatment. In control treatment this only occurred in ‘Pink Champion.’ These cultivar differences were reflected by a higher RUE (8%) in ‘Royal Champion’ in diffuse light treatment compared with control, whereas no effect on RUE was observed in ‘Pink Champion.’ We conclude that the stimulating effect of diffuse light on RUE depends on the stomatal response to temporal PPFD fluctuations, which response is cultivar dependent.

## Introduction

Plants are usually subjected to rapidly alternating periods of sun and shade, which are caused by variable cloud cover, shade from overtopping leaves, leaf flutter, and diurnal rotation of the solar angle (Pearcy, 1990). Consequently, a large fraction of CO_2_ assimilation occurs in fluctuating light conditions, and plant growth strongly correlates with their prevailing light environment and ability to photosynthesize efficiently during light transitions.

Leaves within plant canopies often experience rapid fluctuations in the PPFD, which are caused by the variation of the proportion of direct and diffuse light (Pearcy et al., 2004). Studies have reported that the spatial variation in light intensity in the canopy become less severe under high diffuse/direct ratio condition (Gu et al., 2002; Urban et al., 2007a; Knohl and Baldocchi, 2008; Li et al., 2014a). A homogenous light distribution over the canopy is more efficient for crop photosynthesis, as leaf photosynthesis shows a saturating response to light intensity (Farquhar and Roderick, 2003; Gu et al., 2003; Mercado et al., 2009). Consequently, crop RUE which describes the relation between accumulated plant biomass and intercepted light is higher in diffuse than in direct light (Healey et al., 1998; Sinclair and Muchow, 1999; Alton et al., 2007). Furthermore, the extent of leaf photoinhibition can be alleviated by diffuse light as fewer local peaks in light intensity occur (Urban et al., 2012; Li et al., 2014a), which further improves crop RUE.

Apart from spatial light distribution, increasing diffuse/direct ratio of the incident light results in less variation of temporal light distribution above the plant canopy, which might also play a role for improving crop RUE (Li et al., 2014b). This could be related to the dynamic properties of leaf photosynthesis, which, among others, depend on the dynamic properties of stomatal conductance (*g*_s_). Many studies have investigated dynamic stomatal behavior, i.e., comparing plants from different ecological niches under varying environmental conditions (Tinoco-Ojanguren and Pearcy, 1993; Whitehead and Teskey, 1995; Valladares et al., 1997; Zipperlen and Press, 1997). These studies suggest that the behavior of leaf photosynthetic performance under dynamic light conditions depends on stomatal behavior to a large extent. Changes in stomatal aperture usually take most time to reach a new steady state under transient light conditions (Pearcy et al., 2004; Way and Pearcy, 2012), thus the slow responsiveness of stomatal aperture could limit leaf photosynthesis (Urban et al., 2007b; Kaiser et al., 2014; Lawson and Blatt, 2014). In addition, the strong fluctuations in light intensity might trigger stomatal closure, due to intermittent periods of low light intensity or darkness. Therefore, it can be speculated that stomatal closure that is induced by dynamic light can be alleviated when the incident light above the canopy is made diffuse, because light intensity at a given leaf of the canopy is expected to show smaller temporal fluctuations, this may be beneficial for leaf photosynthesis and thus can improve crop RUE.

Recently diffuse glass has become available that increases the diffuseness of light without affecting light transmission in the greenhouse (Hemming et al., 2008). Several studies have reported that such cover materials increase crop production (or RUE) in the greenhouse, which is mainly attributed to a more homogeneous spatial light distribution within the canopies (Hemming et al., 2007; Markvart et al., 2010; Dueck et al., 2012; Li et al., 2014a). In a recent paper (Li et al., 2014b), we looked at the response of growth and development to diffuse/direct ratio of irradiance in two *Anthurium andreanum* cultivars (‘Pink Champion’ and ‘Royal Champion’). Surprisingly, growth was significantly stimulated by diffuse light in only one of the cultivars (‘Royal Champion’), whereas it was unaffected in the other. A comprehensive yield component analysis pin-pointed RUE of biomass accumulation as the main influential factor to explain this difference. In the current paper, we set out to investigate why RUE was differentially affected by diffuse light between both cultivars.

The aim of this study is to investigate whether the effect of the diffuse/direct ratio of incident light on crop RUE can be explained in terms of dynamic responses of leaf photosynthesis to temporal changes in light intensity. The hypothesis is that the magnitude of the beneficial effect of diffuse light on crop RUE depends on the response of *g*_s_ to dynamic fluctuations of light intensity. To test this hypothesis, a study was conducted in glasshouses covered with clear glass or with diffuse glass which scatters part of the direct solar beam, without affecting light transmission. Two *Anthurium* cultivars (Pink Champion and Royal Champion) were used in this study; According to grower’s experience these two cultivars differed in light sensitivity, with ‘Royal Champion’ being more sensitive to light than ‘Pink Champion.’

## Materials and Methods

### Plant Material and Growth Conditions

Two *Anthurium andreanum* cultivars (‘Pink Champion’ and ‘Royal Champion,’ Anthura, Bleiswijk, The Netherlands) were grown in two Venlo-type glasshouse compartments of 144 m^2^ (15 m × 9.6 m) with a gutter height of 5.5 m at Wageningen UR Greenhouse Horticulture in Bleiswijk (The Netherlands, 52°N, 4.5°E). The two compartments were covered by glass (Guardian Agro, Dudelange, Luxembourg) with 0% haze (clear glass, control) and 71% haze (diffuse glass), respectively. Haze is defined as the percentage of transmitted light that is scattered such that it deviates more than 1.5° from the direction of the incident beam. The hemispherical transmission of PPFD of the glass was 84% for both glass types. The haze factor and hemispherical transmission of the glass were measured in an optical sphere according to ASTM International (2007). The spectral properties of the two glass types were similar in the visible spectrum (400–700 nm) as described by Li et al. (2014b). Three quantum sensors (LI-190, LI-COR, USA) were installed in each of the glasshouse compartments to measure incident PPFD at intervals of 5 min. A standard greenhouse computer (Hogendoorn-Economic, Hogendoorn, Vlaardingen, The Netherlands) was used to control the glasshouse climate (temperature, air humidity and CO_2_ concentration). The experiment included two growing seasons: summer and winter.

Plants, propagated *in vitro*, were raised in a glasshouse by a nursery. When the first flowers had appeared, plants were repotted and moved to experimental glasshouses. The summer growing season started from 6 Apr to 28 Aug 2012. The DLI (mol m^-2^ d^-1^ PAR) was limited to 7.5 mol m^-2^ d^-1^ in both compartments, which was realized by controlling the white sunscreen (LS 16 F Revolux, transmission of 37% and haze factor of 10%, Ludvig Svensson, Kinna, Sweden) and blackout screen (XLS obscural Revolux A/B + B/B, Ludvig Svensson, Kinna, Sweden). These screens were placed in the top of each glasshouse compartment (below gutter height). The white sunscreen was closed when outside global radiation reached 250 W m^-2^. The blackout screen was closed when DLI in the glasshouse compartment reached the threshold value in the afternoon. Opening and closing of screens was controlled by a standard greenhouse computer. Plants were grown on potting soil (30% fine peat + 10% coarse peat + 43% coco peat + 10% bark + 7% perlite) in black plastic pots (12 cm diameter and 11 cm height) on cultivation tables (4 m × 1.8 m) with an automatic ebb/flood irrigation system which supplied irrigation solution once per week in the beginning of the cultivation, while this increased to three times per week from week 10 after the start of the experiment onward. In each compartment, six cultivation tables were used and each table was equally divided into two sections for the cultivation of both cultivars. Plants in the outer two rows of each section were considered as border plants. The starting plant density was 30 plants m^-2^; this was reduced to 20 plants m^-2^ 3 weeks after start of the experiment. After each destructive harvest plants were moved to maintain the same plant density. During the growing season, climate factors were maintained similar in the two treatments except for light condition. Average daily outside global radiation was 16 MJ m^-2^ d^-1^. Inside the greenhouses the average day/night temperature was 25/21°C; relative air humidity was 75/78%; average daytime CO_2_ concentration was 754 μmol mol^-1^; average realized DLI was 7.2 mol m^-2^ d^-1^ in the control and 7.5 mol m^-2^ d^-1^ in the diffuse light treatment.

The winter growing season lasted from September 5, 2012 to April 25, 2013. The experimental set-up was similar as in the summer growing season except that high pressure sodium lamps (Master GreenPower Plus 1000W EL, Philips, Eindhoven, The Netherlands) were applied to supplement incoming radiation when outside global radiation was below 100 W m^-2^, which led to an average realized DLI of 5.6 mol m^-2^ d^-1^ in the control and 5.7 mol m^-2^ d^-1^ in the diffuse light treatment. Average daily outside global radiation during the winter growing season was 6 MJ m^-2^ d^-1^; average day/night temperature inside the greenhouses was 22/19°C; relative air humidity was 75/73%; average daytime CO_2_ concentration was 802 μmol mol^-1^.

### Plant Measurements

During the summer growing season, total plant dry weight (including roots) was destructively determined at 4, 10, 16, 18, and 21 weeks after start of the experiment. Roots were cleaned with water. Plant organs were dried for at least 48 h at 80°C in a ventilated oven. At each destructive measurement, two plants per cultivar were randomly selected from each cultivation table, which resulted in 12 replicates per treatment.

### Canopy Light Interception

Canopy PPFD interception was determined during the summer growing season, which was measured on four overcast days (May 11, June 15, July 17, and August 24) and three clear days (May 23, June 20, and July 25). These days were close to the period when destructive measurements were taken. The measurements were done with a line light probe, in relation to a reference sensor just above the crop (Sunscan, Delta-T, Cambridge, UK). Six measurements were done above as well as below the canopy for each cultivar on each cultivation table. Measurements at the top of the canopy were taken just above the highest leaf, while the bottom measurements were done at pot height. All measurements were carried out between 10 AM and 3 PM during a day.

### Crop Radiation Use Efficiency

Crop RUE was defined as the ratio between the accumulated TDM and the sum of intercepted PPFD during the experimental period, which was estimated by the slope of the linear relationship between the accumulated TDM and the sum of intercepted PPFD. Accumulated TDM was determined by using TDM from each destructive measurement minus TDM determined at the first destructive measurement. For calculating the sum of intercepted PPFD, the time course of fraction of intercepted PPFD was estimated from the four periodic canopy PPFD interception measurements on cloudy days (Eq. 1). These data represent the fraction of intercepted PPFD over the growing season because the fraction of intercepted PPFD measured on clear days was similar as on cloudy days (data not shown). In each treatment, the fraction of intercepted PPFD could be well fitted by a negative exponential curve with number of days after the start of the experiment and reaching a plateau in the end (*r*^2^ = 0.99 for all treatments)

(1) $$I\left(L\right)/I_{0}=1-e^{-ad}$$

where, *I*(*L*)/*I*_o_ is daily fraction of intercepted PPFD, in which *I*(*L*) is PPFD at LAI *L*, *I*_o_ is PPFD at top of canopy; *a* is a coefficient which is derived from the curve fitting; *d* is number of days after start of the experiment.

Daily PPFD intercepted by the canopy was calculated as the product of the interpolated daily fraction of intercepted PPFD [*I*(*L*)*/I*_o_] multiplied by the measured DLI. Integrating the daily PPFD intercepted by the canopy during the designated growing period yielded the sum of intercepted PPFD.

### Net Leaf Photosynthesis

Net leaf photosynthesis (*P*_n_) was measured with a portable gas exchange device (LI-6400XT; LI-COR, Lincoln, NE, USA). During the summer growing season, the transparent leaf chamber (Part No. 6400-08) was used to measure instantaneous *P*_n_ in the control treatment (clear glass) on three clear days for each cultivar. The leaf chamber was horizontally positioned. Three measurements from three plants were taken for each cultivar. In each measurement, *P*_n_, *g*_s_ and incident PPFD were recorded at 1 min intervals on one fully expanded leaf and continuously measured for 2–4 h.

During the winter growing season, instantaneous *P*_n_ was measured on four clear days for each cultivar in both treatments. The measurement procedure was similar as during the summer growing season. Furthermore, instantaneous *P*_n_ of both cultivars were measured on overcast days in the control treatment to further verify their responses to diffuse light. Steady state *P*_n_ light response curves were measured with the portable gas exchange device equipped with a leaf chamber fluorometer (Part No. 6400-40). Six fully expanded leaves were randomly selected from each cultivar in each treatment for this measurement. Selected leaves were adapted at 300 μmol m^-2^ s^-1^ PPFD for 10 min before the measurements were taken. PPFD was varied stepwise in the following sequence: 700, 600, 500, 400, 300, 200, 100, 50, 25, and 0 μmol m^-2^ s^-1^. At each PPFD, the measurements were taken when the *P*_n_ reached steady state.

All measurements were carried out between 9 and 16 h. In the measurement chamber, VPD was kept around 1 kPa, reference CO_2_ concentration was set at 800 μmol m^-2^ s^-1^, leaf temperature at 27 and 25°C for the summer and winter growing season, respectively. These parameters were close to those in the glasshouse.

### Chlorophyll Fluorescence

During the summer growing season, maximum photosystem II (PSII) efficiency (*F*v/*F*m) was measured in week 16 after start of the experiment. Measurements were taken with a portable chlorophyll fluorometer (PAM-2000, Walz, Germany) at five time points (9:00, 11:00, 13:00, 15:00, 17:00 h) on clear days. Red light was used as measuring light (0.5 μmol m^-2^ s^-1^) and saturating flashes (8000 μmol m^-2^ s^-1^). At each time point, four fully expanded leaves of each cultivar in each treatment were randomly selected. A leaf clip holder (DLC-8) was used for dark adaptation for 30 min prior to measurements.

### Statistical Analysis

The non-rectangular hyperbola function (Eq. 2), (Cannell and Thornley, 1998) was fitted to steady state *P*_n_ light response data:

(2) $$P_{n}=\frac{I_{a}+P_{\max}-\sqrt{{\left(I_{a}+P_{\max}\right)}^{2}-4Ia\Theta P_{\max}}}{2\Theta }-R_{d}$$

where, *P*_n_ is net leaf photosynthesis rate (μmol m^-2^ s^-1^), *I* is incident PPFD (μmol m^-2^ s^-1^), *P*_max_ is maximum net leaf photosynthesis rate (μmol m^-2^ s^-1^), *a* is the leaf photosynthetic efficiency (μmol CO_2_ μmol^-1^ photons), Θ is the curvature parameter, and *R*_d_ is dark respiration (μmol m^-2^ s^-1^).

Eq. (2) was also fitted to the measured instantaneous *P*_n_ light response data. The purpose of this fitting was to obtain the standard error of the fit to quantify the variability of the instantaneous *P*_n_ in response to incident PPFD. Comparisons of the standard error of the fit, photosynthesis light response parameters as well as the time course of maximum PSII efficiency (*F*v/*F*m) between treatments were evaluated by analysis of variance (ANOVA), assuming replications in the same glasshouse compartment as being independent. Differences between treatments in RUE were tested by multiple linear regression.

## Results

### Crop RUE and Biomass Production

**FIGURE 1:**
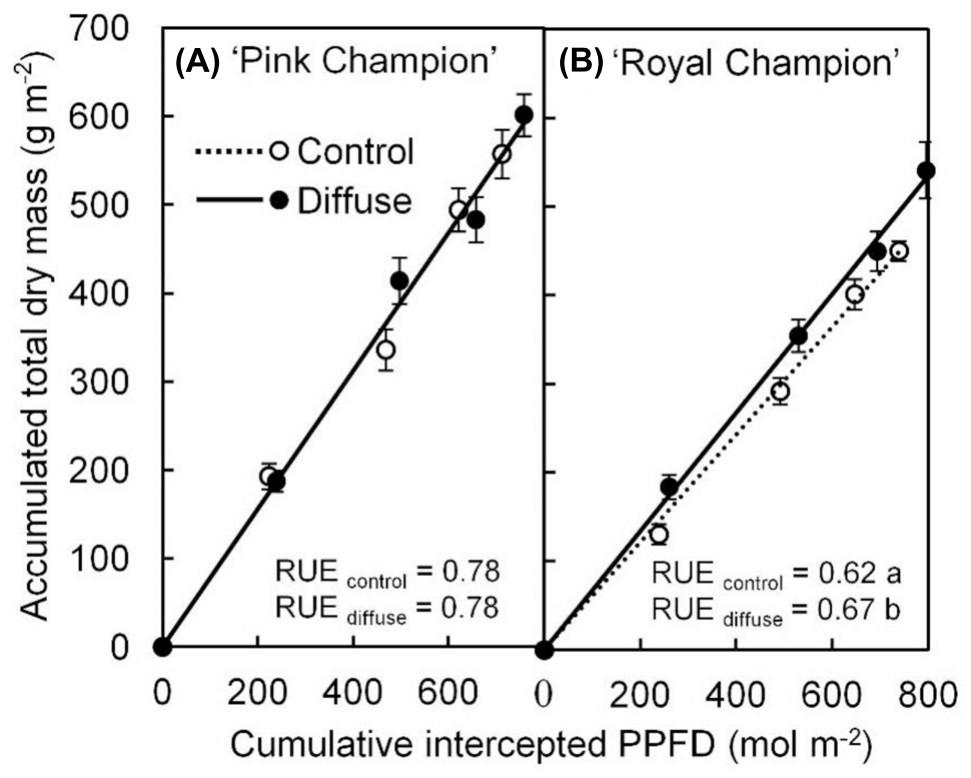
**Relationship between accumulated TDM and cumulative intercepted PPFD for ‘Pink Champion’ (A) and ‘Royal Champion’ (B) in the control and diffuse light treatments.** Solid and dashed lines represent fitted linear relationships for diffuse light and control treatments, respectively. The slope of the fitted linear relationship is the RUE of biomass production (g dry mass mol^-1^ PPFD). Dashed line in ‘Pink Champion’ is obscured by the solid line. Error bars show ±SE (*n* = 12). Letters show statistical significant differences (*P* < 0.05).

The diffuse light treatment stimulated crop RUE by 8% in ‘Royal Champion’ compared with control (**Figure 1B**); while this effect did not occur in ‘Pink Champion’ (**Figure 1A**). Consistent with the increased crop RUE, the diffuse light treatment significantly increased biomass production in ‘Royal Champion’ (*P* = 0.01), but not in ‘Pink Champion’ (*P* = 0.52). For detailed information about biomass production see Li et al. (2014b).

### Steady State *P*_n_

**FIGURE 2:**
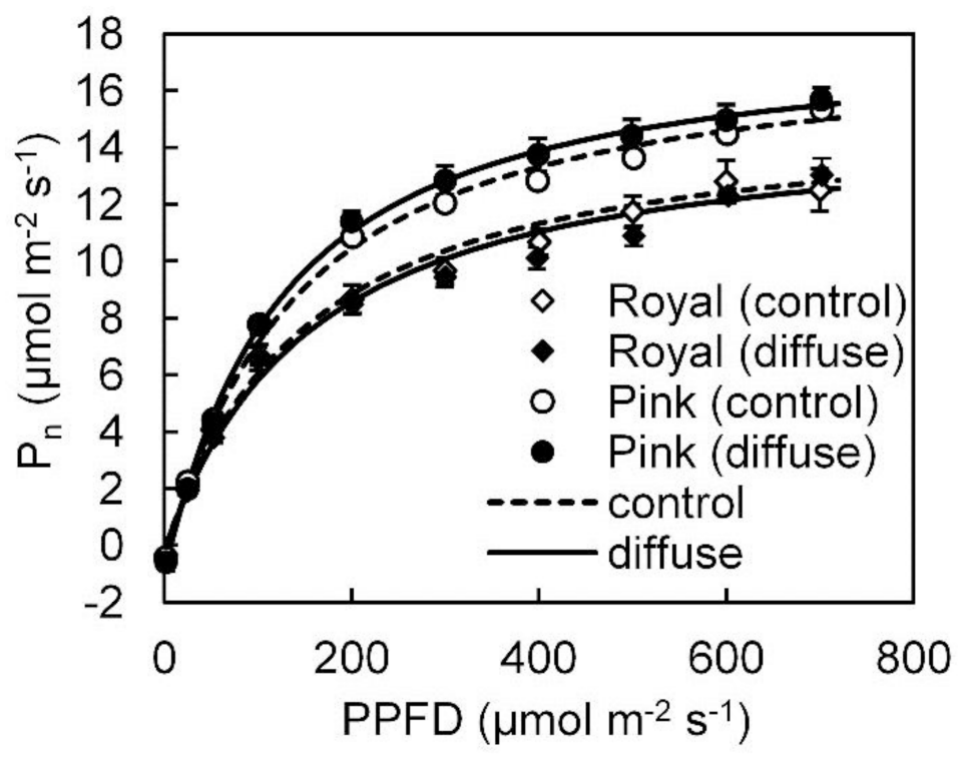
**Steady state net leaf photosynthetic (*P*_n_) light response curves in ‘Pink Champion’ and ‘Royal Champion’ in the control and diffuse light treatments.** The measurements were taken in March 2013 (winter growing season). During the measurements, air temperature, CO_2_ concentration, VPD in the measurement chamber were maintained at 25°C, 800 μmol mol^-1^, and between 0.5–1 kPa, respectively. Lines represent the fit of the non-rectangular hyperbola function (Eq. 2). Error bars show ± SE (*n* = 6).

In the winter growing season, steady state *P*_n_ light response data could be well fitted with a smooth non-rectangular hyperbolic curve (**Figure 2**). Fitted photosynthetic light response curve parameters (i.e., *P*_max_*, a*,Θ*, R*_d_) in both cultivars were not influenced by the diffuse light treatment (see Supplementary Table S1). Leaf photosynthetic capacity in ‘Pink Champion’ was higher than in ‘Royal Champion’ in both treatments (**Figure 2**).

### Stomatal Conductance (*g*_s_) and *P*_n_ Response to Dynamic Light

**FIGURE 3:**
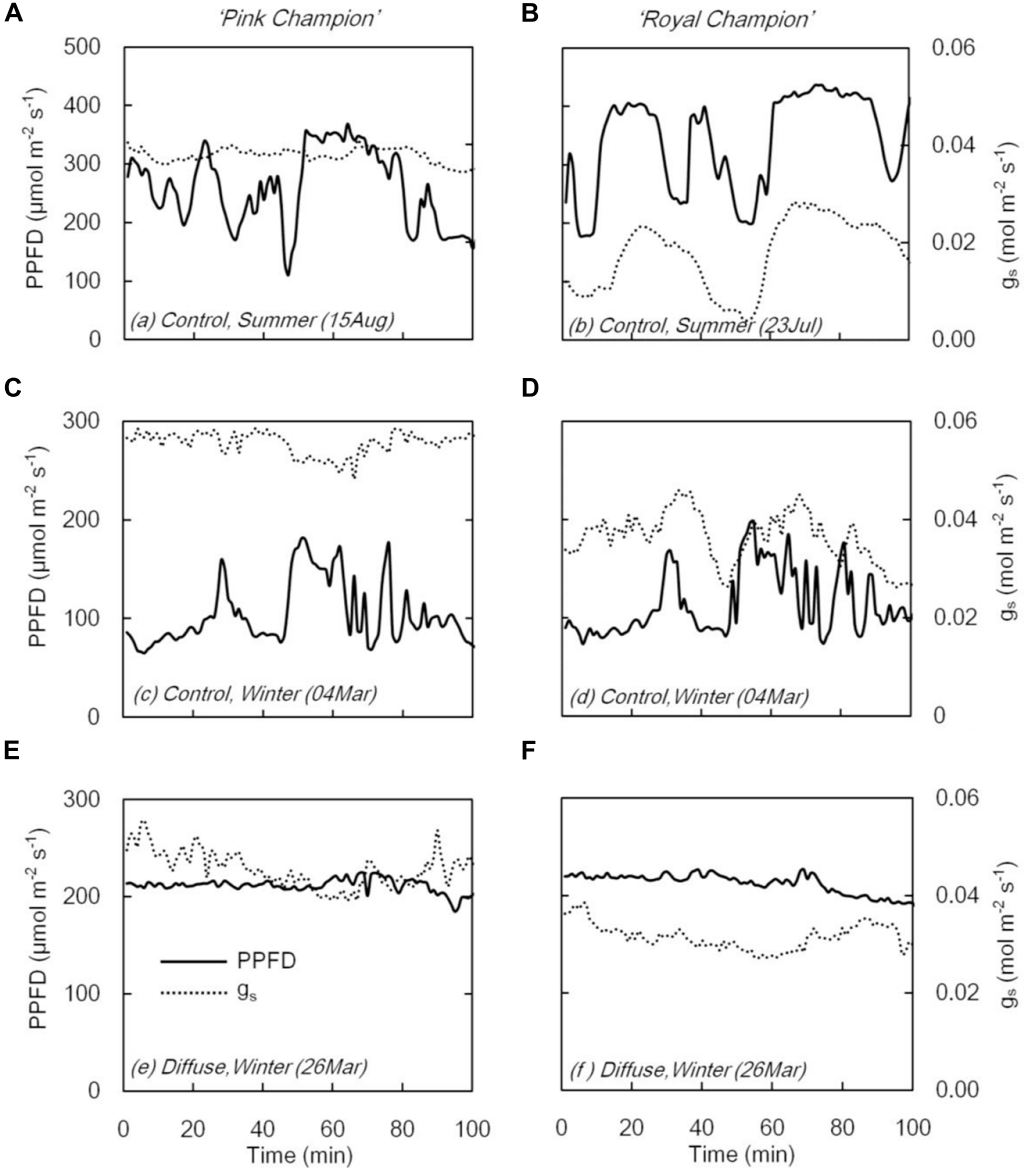
**Stomatal conductance (*g*_s_, dashed line) and PPFD (solid line) in compartment with clear glass (control: A–D) and diffuse glass (E,F). ‘Pink Champion’ is shown in (A,C,E) and ‘Royal Champion’ in (B,D,F).** The measurements were taken at 1 min interval.

On clear days, *g*_s_ in ‘Pink Champion’ varied slightly when incident PPFD was temporally fluctuating in the control treatment (**Figures 3A,C**); while in ‘Royal Champion,’ *g*_s_ strongly responded to the variation of incident PPFD (**Figures 3B,D**). In diffuse light treatment, *g*_s_ in both cultivars showed only minor fluctuations when incident PPFD was relatively constant on clear days (**Figures 3E,F**). Furthermore, *g*_s_ was also relatively constant in both cultivars in the control treatment on overcast days when global radiation was stable and fully diffuse (data not shown).

**FIGURE 4:**
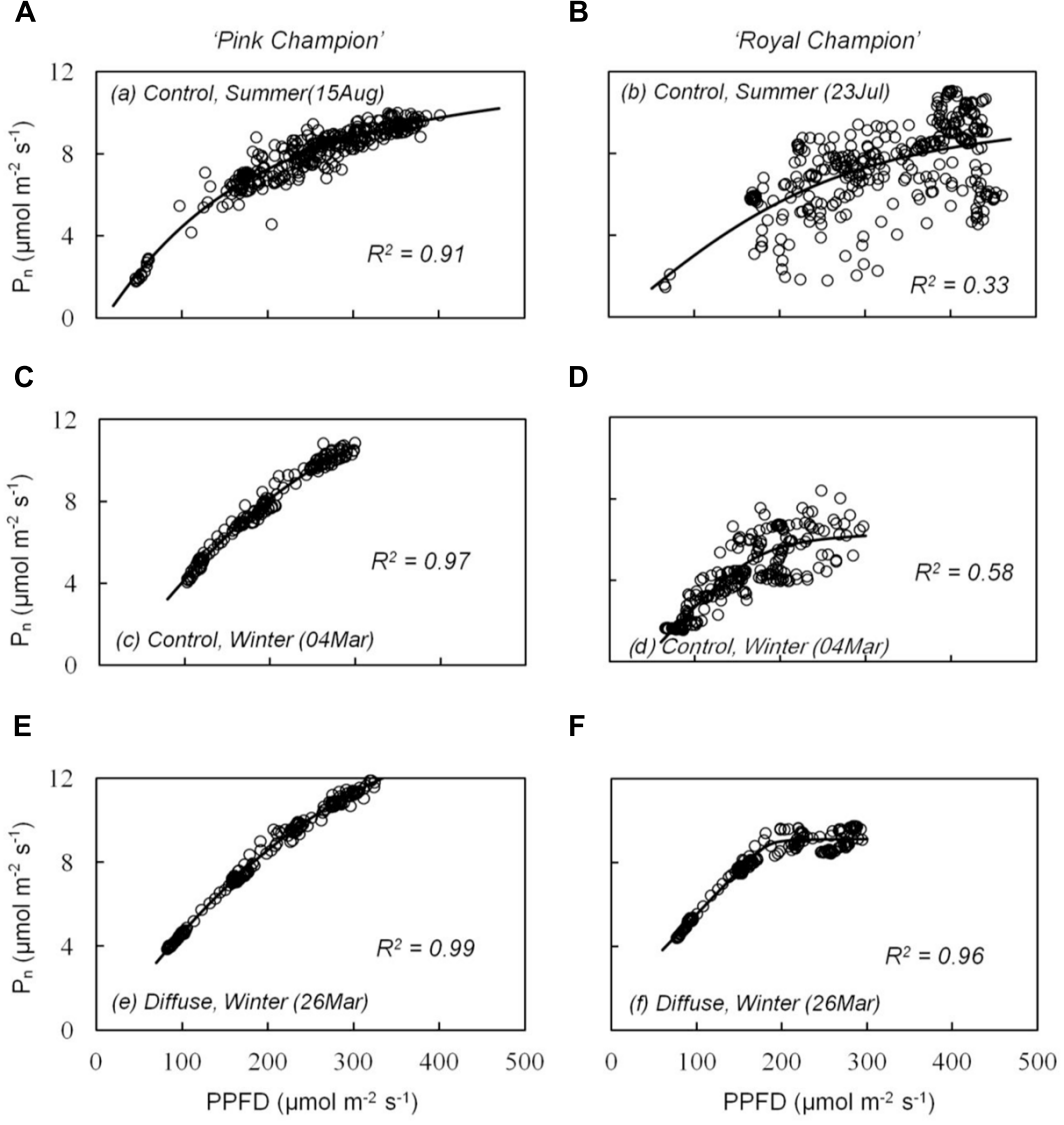
**Relationship between instantaneous net leaf photosynthesis rates (*P*_n_) and transient PPFD in compartment with clear glass (control: A–D) and diffuse glass (E,F). ‘Pink Champion’ is shown in (A,C,E) and ‘Royal Champion’ in (B,D,F).** *P*_n_ and incident PPFD were recorded at 1 min interval on one fully expanded leaf and continuously measured for 2–4 h. Lines represent the best fit of a non-rectangular hyperbola (Eq. 2). Each panel shows an example of one measurement (one replicate) under the specific treatment on clear day.

**FIGURE 5:**
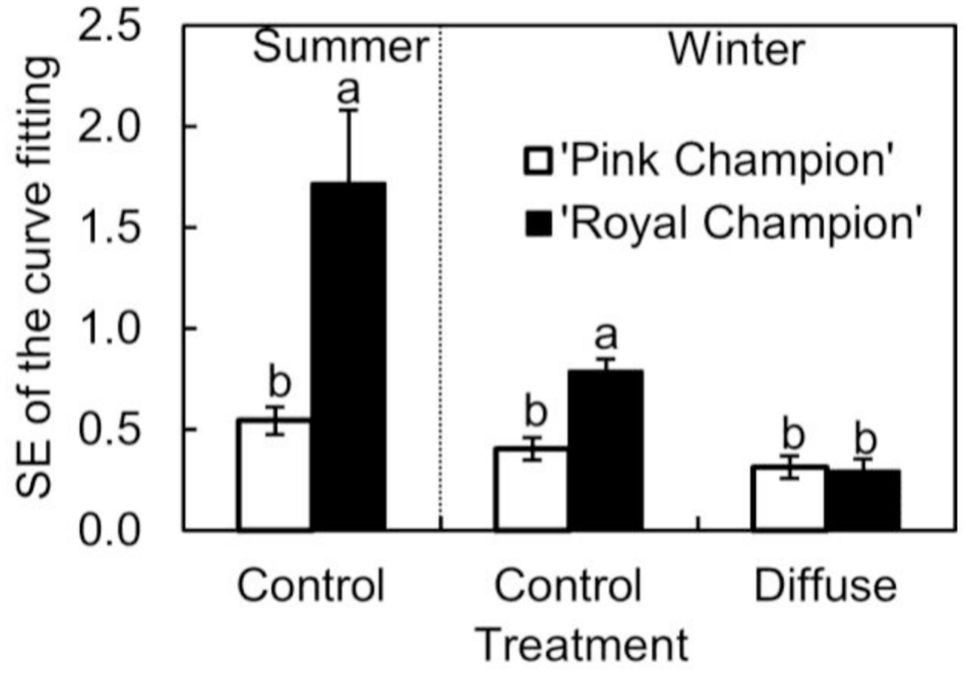
**Standard error of fitting non-rectangular hyperbola to instantaneous net leaf photosynthesis rates as a function of incident PPFD (Eq. 2) in summer growing season in the control treatment (*n* = 3), and in winter growing season in the control as well as in diffuse light treatments (*n* = 4; as see in **Figure 4** which shows one replicate under each specific condition).** Data used for curve fitting in this figure were collected on clear days. Error bars show ± SE. Letters within each growing season show statistical significant differences (*P* < 0.05).

The cultivar difference in *g*_s_ response to the transient light condition resulted in a distinct difference between cultivars in their response of instantaneous *P*_n_. In ‘Pink Champion,’ instantaneous *P*_n_ followed a non-rectangular hyperbolic relationship with incident PPFD on clear days in both treatments (**Figures 4A,C,E**). Similar response patterns were observed for ‘Royal Champion’ in the diffuse light treatment on clear days (**Figure 4F**). However, instantaneous *P*_n_ of ‘Royal Champion’ as a function of incident PPFD showed a highly variable (scattering) response in the control treatment on clear days (**Figures 4B,D**); this phenomenon was more obvious in the summer growing season than in the winter growing season (**Figures 4B,D**). Differences in the scattering of the instantaneous *P*_n_ as a function of incident PPFD were quantified by comparing the standard errors of best-fits of the data to non-rectangular hyperbolic curves, which showed that standard errors in ‘Royal Champion’ were significantly larger than in ‘Pink Champion’ in the control treatment (**Figure 5**). In the diffuse light treatment, standard errors were low and no significant difference between cultivars was found (*P* = 0.824).

## Discussion

The spatial distribution of diffuse light within the canopy is more homogeneous than that of direct light, which results in an enhanced canopy photosynthesis (Spitters, 1986; Healey et al., 1998; Roderick et al., 2001; Li et al., 2014a). At the leaf level, diffusivity of the incident light above the canopy also strongly reduces the temporal variation in light intensity (Li et al., 2014b), which mainly due to diffuse light minimized the effects of local shade by construction parts, and produced less variation of temporal light distribution above canopies. Accordingly, we speculate that the temporal variation of light intensity within the canopy was also significantly reduced under diffuse light. To our knowledge, we are the first to show that the effects of an increased ratio of diffuse/direct light (by diffuse glass cover) on crop RUE are mediated by a decrease in temporal variation of light intensity at leaf level. We explain how differences in dynamic stomatal properties between varieties can modulate the response of crop RUE to diffusivity of incident light.

### What could be the Potential Explanation for the Stimulating Effect of Diffuse Light on RUE?

Radiation use efficiency provides the measure that directly reflects the efficiency of a crop to utilize the radiant energy for producing biomass, which is usually determined by an integration of many factors, for instance, leaf photosynthetic capacity, plant growth environment, canopy structure as well as nutrient condition (Sinclair and Muchow, 1999). In this study, increasing the diffuse/direct ratio of incident light resulted in 8% increase in RUE in ‘Royal Champion’ (**Figure 1B**). The stimulating effect of diffuse light on RUE has been found in many studies on a variety of plant species and was mainly explained by a more homogeneous vertical light distribution within the canopy (Sinclair et al., 1992; Healey et al., 1998; Alton et al., 2007; Zhang et al., 2011). We observed that the fraction of light intercepted by the canopy as a function of LAI was not affected by treatments in this study (Li et al., 2014b), indicating that the vertical light distribution was not affected. The absence of such effect can be explained by the fact that anthurium pot-plants have a short and compact canopy structure, which is less responsive to scattering of the light (Li et al., 2014b). Additionally, shading screens were applied in both treatments, which already transformed a portion of direct solar light into diffuse (10%). Li et al. (2014a) showed that diffuse light is also more homogeneously distributed in the horizontal plane within a tomato canopy compared to direct light. Although we did not investigate the horizontal light distribution within the canopy in this study, we speculate that its effect on crop RUE was small, since approximately 80% of the time incident PPFD was below 300 μmol m^-2^ s^-1^ in both treatments during the summer growing season, implying that *P*_n_ during a large part of the growing season was likely in the linear range of the photosynthesis light response curve (**Figure 2**). Therefore, it seems likely that the homogeneity of the spatial light distribution within the canopy did not play an important role for improving crop RUE in this study.

Radiation use efficiency could also be affected by other environmental factors such as temperature (Andrade et al., 1993), relative humidity (Stockle and Kiniry, 1990), water availability (Jamieson et al., 1995), and nutrient condition (Allen et al., 2005). However, these factors were kept similar in both treatments. Due to relative humidity highly modulate the response of stomatal, we checked the daily variation of relative humidity in both treatments on fully clear days and could not found large differences (Supplementary Figure S1). This eliminated the potential effect of these factors as explanations for the differences in RUE between treatments. Although shade plants are particularly susceptible to photoinhibition when exposed to high light (Long et al., 1994) or sunflecks (Powles and Bjorkman, 1981), *F*v/*F*m was consistently around 0.8 during a clear day in this study (see Supplementary Figure S2), indicating a well-functioning photosynthesis apparatus.

Radiation use efficiency can be improved via increases in *P*_n_ (Sinclair and Muchow, 1999). Steady state *P*_n_ showed similar response to PPFD in both treatments and both cultivars with a smooth rectangular hyperbola curve during the winter experiment (**Figure 2**; Supplementary Table S1). Similarly, steady state *P*_n_ was also not influenced by the treatments in the summer experiment (Li et al., 2014b). Therefore, it is clear that steady state *P*_n_ did not contribute to the stimulating effect of diffuse light on crop RUE. A higher leaf photosynthetic capacity was observed in ‘Pink Champion’ than in ‘Royal Champion’ (**Figure 2**), which correlates with higher RUE in ‘Pink Champion’ than in ‘Royal Champion’ (**Figure 1**).

### Effects of Diffuse Light on RUE Depends on the Dynamic Properties of Leaf Photosynthesis

Despite the absence of an effect of the diffuse light treatment on the light response of steady state *P*_n_ in both cultivars, the effect of the diffuse light treatment on the light response of instantaneous *P*_n_ differed between cultivars (**Figure 4**). Plants usually experience frequent variations in light intensity, which are of particular importance because interactions between stomatal and photosynthesis responses may result in prolonged periods where a steady state is not achieved (Knapp and Smith, 1990b).

When incident PPFD fluctuated, the two cultivars showed different responses with respect to *g*_s_ (**Figure 3**). In ‘Pink Champion,’ *g*_s_ remained relatively constant in both treatments irrespective of the short term fluctuations in incident PPFD (**Figures 3A,C,E**). This is likely due to the slow responses of guard cells to changes in PPFD or the initial *g*_s_ was already at its maximum level even at low PPFD (Knapp and Smith, 1990b). In ‘Royal Champion’ *g*_s_ varied considerably with fluctuations in PPFD (**Figures 3B,D**). This behavior may reflect the intrinsic characteristics of stomata that might maximize water use efficiency at the expense of carbon gain (Knapp and Smith, 1990a; Lawson, 2009; Vico et al., 2011). The strong fluctuations in *g*_s_ in ‘Royal Champion’ were not observed in the diffuse light treatment where the temporal changes of incident PPFD were smaller on clear days (**Figure 3F**).

The response in stomatal movement usually lags behind the response of photosynthesis in fluctuating light (Barradas and Jones, 1996; Lawson and Blatt, 2014). Therefore, *g*_s_ can limit leaf photosynthesis under dynamic light conditions, which was indicated by the strong scattering of the instantaneous *P*_n_ when plotted as a function of incident PPFD in ‘Royal Champion’ in the control treatment on clear days (**Figures 4B,D** and **5**). In ‘Pink Champion,’ instantaneous *P*_n_ in many cases approached the values of steady state *P*_n_, when compared at similar PPFD. This indicates that in ‘Pink Champion’ *g*_s_ did not impose a limitation to leaf photosynthesis under dynamic light conditions because of a smaller response of *g*_s_ to variations of incident PPFD.

Incident PPFD with less temporal variation may smooth the fluctuations in *g*_s_, especially in those plants with stomata that react strongly to fluctuations in PPFD. Consistent with this hypothesis, the diffuse light treatment reduced the temporal variation in the incident PPFD (Li et al., 2014b), and consequently alleviated stomatal limitation to leaf photosynthesis in ‘Royal Champion.’ This was illustrated by the reduced scattering of instantaneous *P*_n_ as a function of incident PPFD in the diffuse light treatment on clear days (**Figures 4F** and **5**); a similar phenomenon was observed in the control treatment on cloudy days when global radiation was fully diffuse (data not show). Therefore, we conclude that less stomatal limitation (i.e., small variation in *g*_s_) in diffuse light treatment was beneficial for leaf and canopy photosynthesis in ‘Royal Champion’ and consequently improved RUE. This effect did not occur in ‘Pink Champion,’ mainly because of a smaller response of *g*_s_ to the fluctuations of incident PPFD.

To further verify our finding, the non-rectangular hyperbola function (Eq. 2) was fitted to instantaneous leaf photosynthesis light response data in the diffuse light treatment on clear days. These fitted curves were compared to the measured instantaneous leaf photosynthesis light response data in the control treatment on clear days. From this comparison, the cumulative estimated leaf photosynthesis in the diffuse light treatment were 21 and 6% higher than in the control treatment for ‘Royal Champion’ and ‘Pink Champion,’ respectively (see Supplementary Figure S3). Considering the instantaneous leaf photosynthesis was measured during clear periods, while the growing season was not always on clear period, and diffuse radiation roughly accounted for 50% of global radiation in the Netherlands (Li et al., 2014a); moreover, instantaneous leaf photosynthesis was measured only in top leaves. Therefore, these differences of 21 and 6% in cumulative leaf photosynthesis could comparable to the 8 and 0% increases in RUE in diffuse light treatment for ‘Royal Champion’ and ‘Pink Champion,’ respectively. This further indicates that diffuse light reduced the limitation on leaf photosynthesis for the plants with stomata that react strongly to fluctuations in PPFD.

The scattering response of instantaneous *P*_n_ as a function of incident PPFD for ‘Royal Champion’ in the control treatment was more pronounced in the summer growing season than in the winter growing season (**Figures 4B,D** and **5**). This could be a consequence of the higher fraction of direct light on clear days entering the greenhouse in the summer season compared to the winter season. Therefore, it can be speculated that the stimulating effect of diffuse light on crop RUE in areas with a seasonal climate is more pronounced in the summer season than in the winter season.

In the experiment, anthurium pot-plants were grown at 800 μmol mol^-1^ CO_2_, as this is commonly applied for cultivating shade tolerant pot plants. Under such CO_2_ condition, photosynthesis rates of these two cultivars were still limited by CO_2_ concentration as shown by the continuous strong increase in leaf photosynthesis rates with raising CO_2_ concentration in the range of 50–1600 μmol mol^-1^ (see Supplementary Figure S4).

## Conclusion

Increasing the diffuse/direct ratio of the incident PPFD reduces the temporal variation of incident PPFD at leaf level. In cultivars where stomata respond strongly to fluctuations of PPFD, transient rates of *P*_n_ and subsequently RUE increased when the ratio of diffuse/direct PPFD was increased. In such cultivars, *g*_s_ becomes relatively constant and less limiting for *P*_n_ when the ratio of diffuse/direct PPFD increases. For cultivars with relatively insensitive stomata to the fluctuations of PPFD, the effect of the homogeneous temporal distribution of PPFD on RUE was non-existing. We conclude that additional to previously reported benefits of diffuse light associated with improved spatial light distribution, the stimulating effect of diffuse light on crop RUE can also depend on the dynamic response of *g*_s_ to incident PPFD at leaf level.

## Author Contributions

TL carried out the measurements, data analysis, and drafted the manuscript. JK, LM, and EH made substantial contributions to conception and experiment design, and critically revised the manuscript. EK made dynamic leaf photosynthesis measurement. FN made crop management.

## Conflict of Interest Statement

The authors declare that the research was conducted in the absence of any commercial or financial relationships that could be construed as a potential conflict of interest.

## Abbreviations

DLI: daily light integral
*F*v/*F*m: maximum photosystem II (PSII) efficiency
*g*_s_: stomatal conductance
LAI: leaf area index
*P*_n_: net leaf photosynthesis rate
PAR: photosynthetic active radiation
PPFD: photosynthetic photon flux density
RUE: radiation use efficiency
TDM: total dry mass
VPD: vapor pressure deficit

## References

[B1] Allen C. B. Will R. E. McGarvey R. C. Coyle D. R. Coleman M. D. (2005). Radiation-use efficiency and gas exchange responses to water and nutrient availability in irrigated and fertilized stands of sweetgum and sycamore. *Tree Physiol.* 25 191–200. 10.1093/treephys/25.2.191 15574400

[B2] Alton P. B. North P. R. Los S. O. (2007). The Impact of diffuse sunlight on canopy light use efficiency, gross photosynthetic product and net ecosystem exchange in three forest biomes. *Glob. Change Biol.* 13 776–787. 10.1111/j.1365-2486.2007.01316.x

[B3] Andrade F. H. Uhart S. A. Cirilo A. (1993). Temperature affects radiation use efficiency in maize. *Field Crops Res.* 32 17–25. 10.1016/0378-4290(93)90018-I

[B4] Barradas V. L. Jones H. G. (1996). Responses of CO2 assimilation to changes in irradiance: laboratory and field data and a model for beans (*Phaseolus vulgaris* L.). *J. Exp. Bot.* 47 639–645. 10.1093/jxb/47.5.639

[B5] Cannell M. G. R. Thornley J. H. M. (1998). Temperature and CO2 responses of leaf and canopy photosynthesis: a clarification using the non-rectangular hyperbola model of photosynthesis. *Ann. Bot.* 82 883–892. 10.1006/anbo.1998.0777

[B6] Dueck T. A. Janse J. Li T. Kempkes F. Eveleens B. (2012). Influence of diffuse glass on the growth and production of tomato. *Acta Hortic.* 956 75–82. 10.17660/ActaHortic.2012.956.6

[B7] Farquhar G. D. Roderick M. L. (2003). Pinatubo, diffuse light, and the carbon cycle. *Science* 299 1997–1998. 10.1126/science.1080681 12663904

[B8] Gu L. Baldocchi D. Verma S. B. Black T. A. Vesala T. Falge E. M. (2002). Advantages of diffuse radiation for terrestrial ecosystem productivity. *J. Geophys. Res. D Atmos.* 107 4050 10.1029/2001JD001242

[B9] Gu L. Baldocchi D. D. Wofsy S. C. Munger J. W. Michalsky J. J. Urbanski S. P. (2003). Response of a deciduous forest to the Mount Pinatubo eruption: enhanced photosynthesis. *Science* 299 2035–2038. 10.1126/science.1078366 12663919

[B10] Healey K. D. Rickert K. G. Hammer G. L. Bange M. P. (1998). Radiation use efficiency increases when the diffuse component of incident radiation is enhanced under shade. *Aust. J. Agric. Res.* 49 665–672. 10.1071/A97100

[B11] Hemming S. Dueck T. A. Janse J. van Noort F. (2007). The effect of diffuse light on crops. *Acta Hortic.* 801 1293–1300.

[B12] Hemming S. Mohammadkhani V. Dueck T. A. (2008). Diffuse greenhouse covering materials-material technology, measurements and evaluation of optical properties. *Acta Hortic.* 797 469–475. 10.17660/ActaHortic.2008.797.68

[B13] International A. S. T. M. (2007). *Standard Test Method for Haze and Luminous Transmittance Of Transparent Plastics, D1003-07*. West Conshohocken, PA: ASTM International.

[B14] Jamieson P. D. Martin R. J. Francis G. S. Wilson D. R. (1995). Drought effects on biomass production and radiation-use efficiency in barley. *Field Crops Res.* 43 77–86. 10.1016/0378-4290(95)00042-O

[B15] Kaiser E. Morales A. Harbinson J. Kromdijk J. Heuvelink E. Marcelis L. F. M. (2014). Dynamic photosynthesis in different environmental conditions. *J. Exp. Bot.* 66 2415–2426. 10.1093/jxb/eru406 25324402

[B16] Knapp A. K. Smith W. K. (1990a). Contrasting stomatal responses to variable sunlight in two subalpine herbs. *Am. J. Bot.* 77 226–231. 10.2307/2444644 30139080

[B17] Knapp A. K. Smith W. K. (1990b). Stomatal and photosynthetic responses to variable sunlight. *Physiol. Plant.* 78 160–165. 10.1111/j.1399-3054.1990.tb08731.x

[B18] Knohl A. Baldocchi D. D. (2008). Effects of diffuse radiation on canopy gas exchange processes in a forest ecosystem. *J. Geophys. Res. G Biogeosci.* 113 G02023.

[B19] Lawson T. (2009). Guard cell photosynthesis and stomatal function. *New Phytol.* 181 13–34. 10.1111/j.1469-8137.2008.02685.x 19076715

[B20] Lawson T. Blatt M. R. (2014). Stomatal size, speed, and responsiveness impact on photosythesis and water use efficiency. *Plant Physiol.* 164 1556–1570. 10.1104/pp.114.237107 24578506 PMC3982722

[B21] Li T. Heuvelink E. Dueck T. A. Janse J. Gort G. Marcelis L. F. M. (2014a). Enhancement of crop photosynthesis by diffuse light: quantifying the contributing factors. *Ann. Bot.* 114 145–156. 10.1093/aob/mcu071 24782436 PMC4071095

[B22] Li T. Heuvelink E. Van Noort F. Kromdijk J. Marcelis L. F. M. (2014b). Responses of two *Anthurium* cultivars to high daily integrals of diffuse light. *Sci. Hortic.* 179 306–313. 10.1016/j.scienta.2014.09.039

[B23] Long S. P. Humphries S. Falkowski P. G. (1994). Photoinhibition of photosynthesis in nature. *Annu. Rev. Plant Biol.* 45 633–662. 10.1146/annurev.pp.45.060194.003221

[B24] Markvart J. Rosenqvist E. Aaslyng J. M. Ottosen C. O. (2010). How is canopy photosynthesis and growth of chrysanthemums affected by diffuse and direct light? *Eur. J. Hortic. Sci.* 75 253–258.

[B25] Mercado L. M. Bellouin N. Sitch S. Boucher O. Huntingford C. Wild M. (2009). Impact of changes in diffuse radiation on the global land carbon sink. *Nature* 458 1014–1017. 10.1038/nature07949 19396143

[B26] Pearcy R. W. (1990). Sunflecks and photosynthesis in plant canopies. *Annu. Rev. Plant Biol.* 41 421–453. 10.1146/annurev.pp.41.060190.002225

[B27] Pearcy R. W. Krall J. P. Sassenrath-Cole G. F. (2004). “Photosynthesis in fluctuating light environments,” in *Photosynthesis and the Environment*, ed. Baker N. R. (Dordrecht: Springer), 321–346.

[B28] Powles S. B. Bjorkman O. (1981). *Leaf Movement in the Shade Species Oxalis Oregana. II. Role in Protection Against Injury by Intense Light.* Washington, DC: Year Book-Carnegie Institution of Washington.

[B29] Roderick M. L. Farquhar G. D. Berry S. L. Noble I. R. (2001). On the direct effect of clouds and atmospheric particles on the productivity and structure of vegetation. *Oecologia* 129 21–30. 10.1007/s004420100760 28547064

[B30] Sinclair T. R. Muchow R. C. (1999). Radiation use efficiency. *Adv. Agron.* 65 215–265. 10.1016/S0065-2113(08)60914-1

[B31] Sinclair T. R. Shiraiwa T. Hammer G. L. (1992). Variation in crop radiation use efficiency with increased diffuse radiation. *Crop Sci.* 32 1281–1284. 10.2135/cropsci1992.0011183X003200050043x

[B32] Spitters C. J. T. (1986). Separating the diffuse and direct component of global radiation and its implications for modeling canopy photosynthesis Part II. Calculation of canopy photosynthesis. *Agric. For. Meteorol.* 38 231–242. 10.1016/0168-1923(86)90061-4

[B33] Stockle C. O. Kiniry J. R. (1990). Variability in crop radiation-use efficiency associated with vapor-pressure deficit. *Field Crops Res.* 25 171–181. 10.1016/0378-4290(90)90001-R

[B34] Tinoco-Ojanguren C. Pearcy R. W. (1993). Stomatal dynamics and its importance to carbon gain in two rainforest *Piper* species. *Oecologia* 94 395–402. 10.1007/BF00317115 28313677

[B35] Urban O. Janous D. Acosta M. Czerny R. MarkovA I. NavrATil M. (2007a). Ecophysiological controls over the net ecosystem exchange of mountain spruce stand. Comparison of the response in direct vs. diffuse solar radiation. *Glob. Change Biol.* 13 157–168. 10.1111/j.1365-2486.2006.01265.x

[B36] Urban O. Košvancová M. Marek M. V. Lichtenthaler H. K. (2007b). Induction of photosynthesis and importance of limitations during the induction phase in sun and shade leaves of five ecologically contrasting tree species from the temperate zone. *Tree Physiol.* 27 1207–1215. 10.1093/treephys/27.8.1207 17472946

[B37] Urban O. Klem K. Aè A. Havránková K. Holišová P. Navrátil M. (2012). Impact of clear and cloudy sky conditions on the vertical distribution of photosynthetic CO2 uptake within a spruce canopy. *Funct. Ecol.* 26 46–55. 10.1111/j.1365-2435.2011.01934.x

[B38] Valladares F. Allen M. T. Pearcy R. W. (1997). Photosynthetic responses to dynamic light under field conditions in six tropical rainforest shrubs occuring along a sight gradient. *Oecologia* 111 505–514. 10.1007/s004420050264 28308111

[B39] Vico G. Manzoni S. Palmroth S. Katul G. (2011). Effects of stomatal delays on the economics of leaf gas exchange under intermittent light regimes. *New Phytol.* 192 640–652. 10.1111/j.1469-8137.2011.03847.x 21851359

[B40] Way D. A. Pearcy R. W. (2012). Sunflecks in trees and forests: from photosynthetic physiology to global change biology. *Tree Physiol.* 32 1066–1081. 10.1093/treephys/tps064 22887371

[B41] Whitehead D. Teskey R. O. (1995). Dynamic response of stomata to changing irradiance in loblolly pine (*Pinus taeda* L.). *Tree Physiol.* 15 245–251. 10.1093/treephys/15.4.245 14965964

[B42] Zhang M. Yu G. R. Zhuang J. Gentry R. Fu Y. L. Sun X. M. (2011). Effects of cloudiness change on net ecosystem exchange, light use efficiency, and water use efficiency in typical ecosystems of China. *Agric. For. Meteorol.* 151 803–816. 10.1016/j.agrformet.2011.01.011

[B43] Zipperlen S. W. Press M. C. (1997). Photosynthetic induction and stomatal oscillations in relation to the light environment of two dipterocarp rain forest tree species. *J. Ecol.* 85 491–503. 10.2307/2960572

