# Supplementary material for: Effects of Diffuse Light on Radiation Use Efficiency of Two Anthurium Cultivars Depend on the Response of Stomatal Conductance to Dynamic Light Intensity
Source: Front Plant Sci. 2016 Feb 4;7:56. doi: 10.3389/fpls.2016.00056 (PMC4740510; doi:10.3389/fpls.2016.00056)
Supplement: Supplementary file 1 [file Data_Sheet_1.DOCX]

**Supplementary data**

| **Table S1.** Net leaf photosynthetic light response curve parameters in ‘Pink Champion’ and ‘Royal Champion’ in the control and diffuse light treatments (n = 6). These are fitted from measured Net leaf photosynthetic light response curve [eqn (2)]. | | | | |
| --- | --- | --- | --- | --- |
|  |  |  |  |  |
| Treatments | *P_max_*_,_  (µmol m^-2^ s^-1^) | *α* (µmol CO_2_ µmol^-1^ photons) | *Ө* | *R_d_* (µmol m^-2^ s^-1^) |
|  |  |  |  |  |
| ‘Royal Champion’ | | | | |
| Control | 16.0 | 0.10 | 0.20 | -0.16 |
| Diffuse | 15.5 | 0.09 | 0.20 | -0.16 |
| ‘Pink Champion’ | | | | |
| Control | 17.4 | 0.13 | 0.20 | -0.65 |
| Diffuse | 18.0 | 0.13 | 0.20 | -0.80 |
| Statistical analysis showed that net leaf photosynthetic light response curve parameters were not significantly different between treatments. | | | | |

**
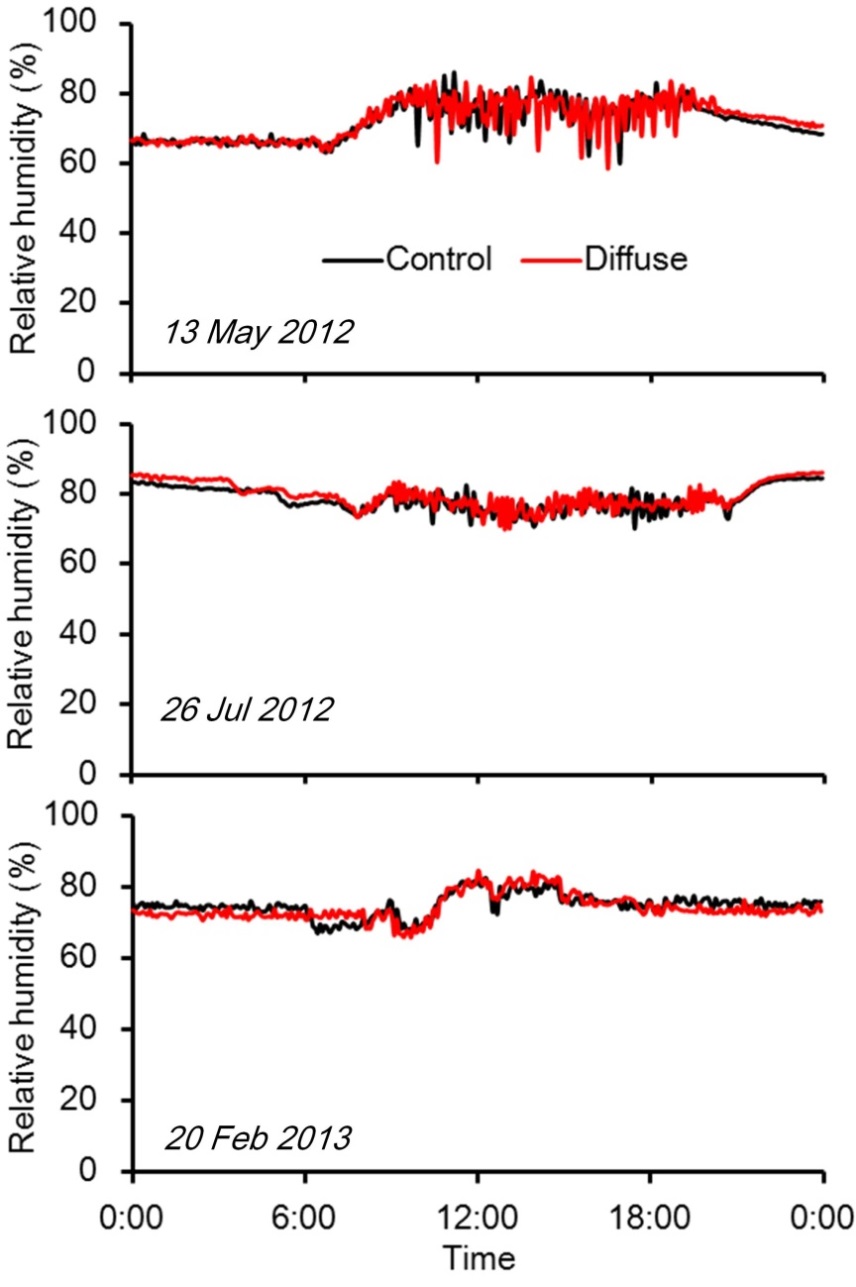
**

**Fig. S1.** Relative air humidity in the control and diffuse light treatments on three clear days (as an example). Data were recorded by a standard greenhouse computer at 5 minutes interval.


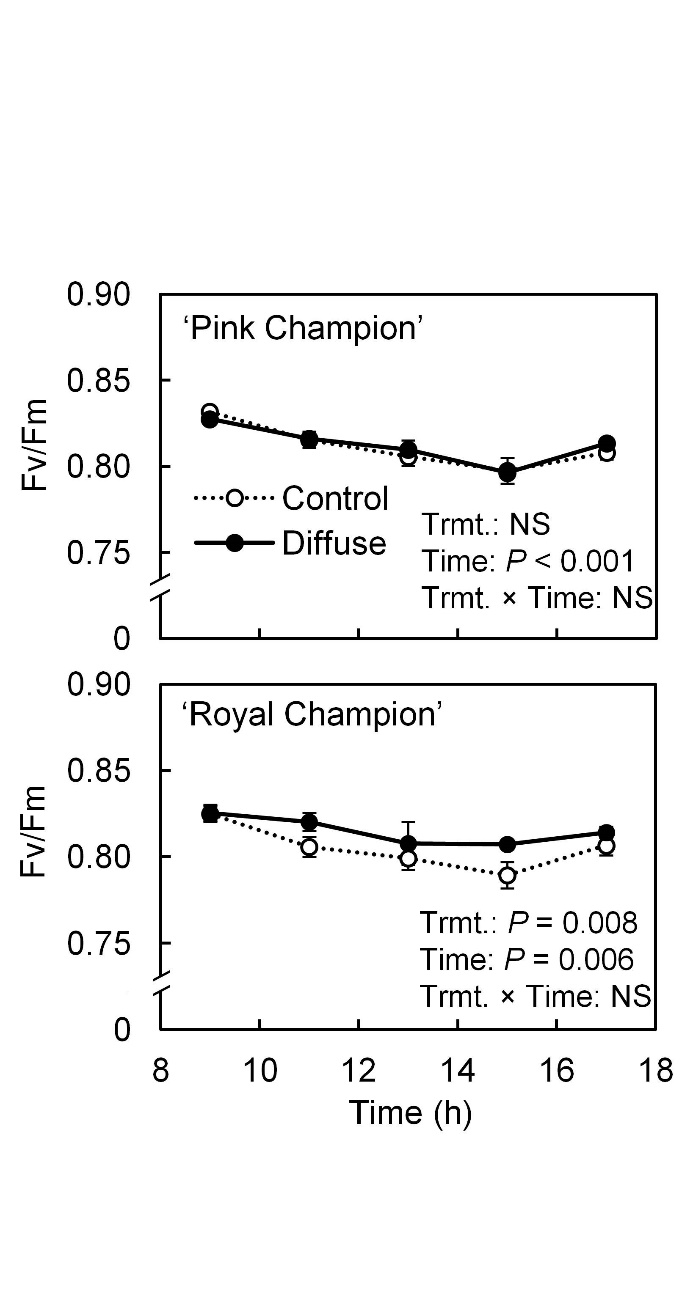


**Fig. S2.** Maximum PSII efficiency (Fv/Fm) in ‘Pink Champion’ and ‘Royal Champion’ in the control and diffuse light treatment on a clear day in the summer growing season (26 July 2012: for PPFD see Fig 1). Error bars show ± SE (n = 4). Two-way ANOVA with treatment (Trmt.) and time as independent variables and their interaction (Trmt. × Time) for each dependent variable is shown in each cultivar. NS: not significant (P ≥ 0.05).


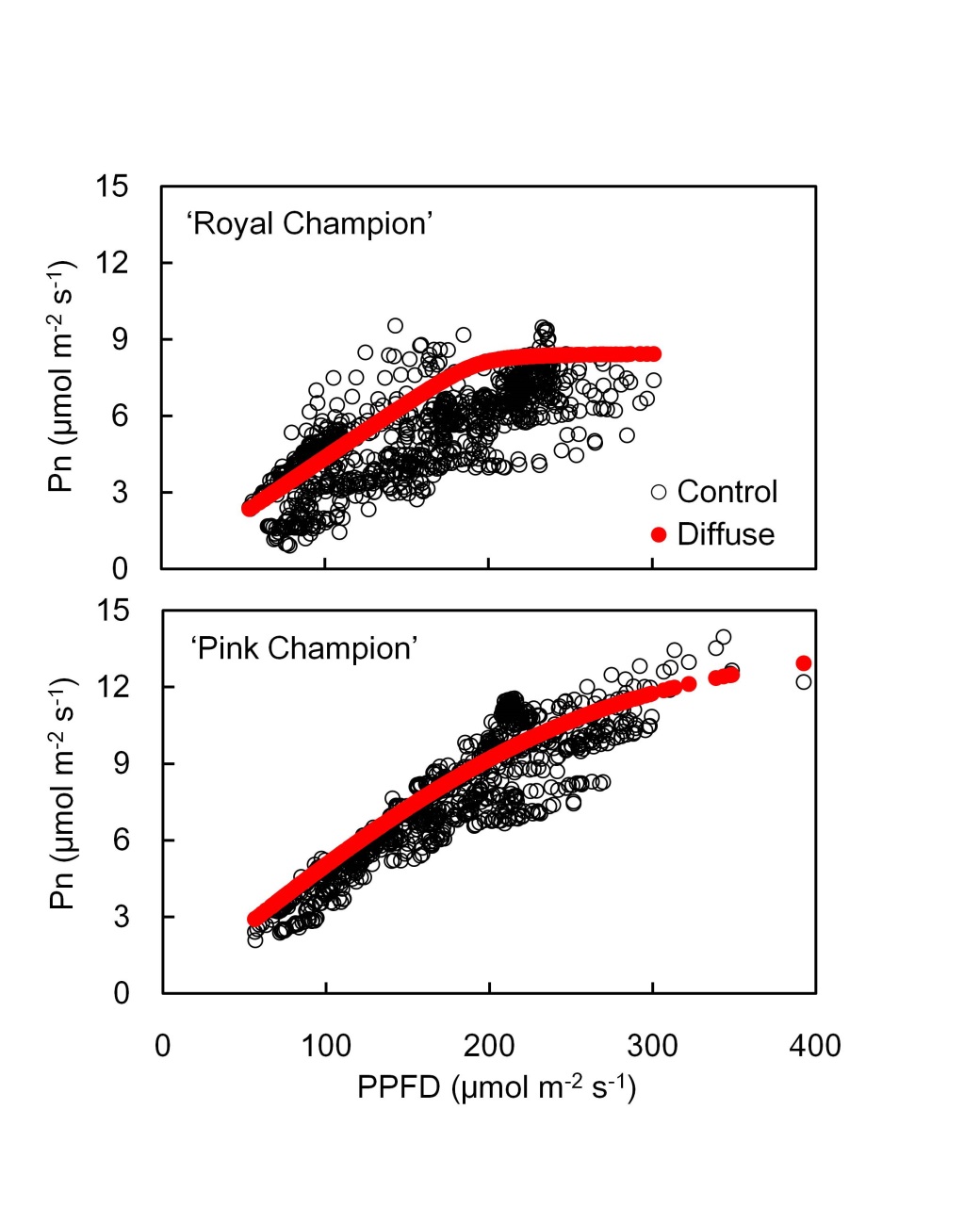


Fig. S3. Comparison of leaf photosynthesis in the control and diffuse light treatment. Red symbol represents photosynthesis light response curve in the diffuse light treatment, which is the non-rectangular hyperbola function [eqn (2)] fitted to measured instantaneous leaf photosynthesis light response data in the diffuse light treatment on clear days (n=4). Black symbol represents measured instantaneous leaf photosynthesis light response data in the control treatment on clear days (n=4). From this comparison, the cumulative leaf photosynthesis in the diffuse light treatment were 21% and 6% higher than in the control treatment for ‘Royal Champion’ and ‘Pink Champion’, respectively.


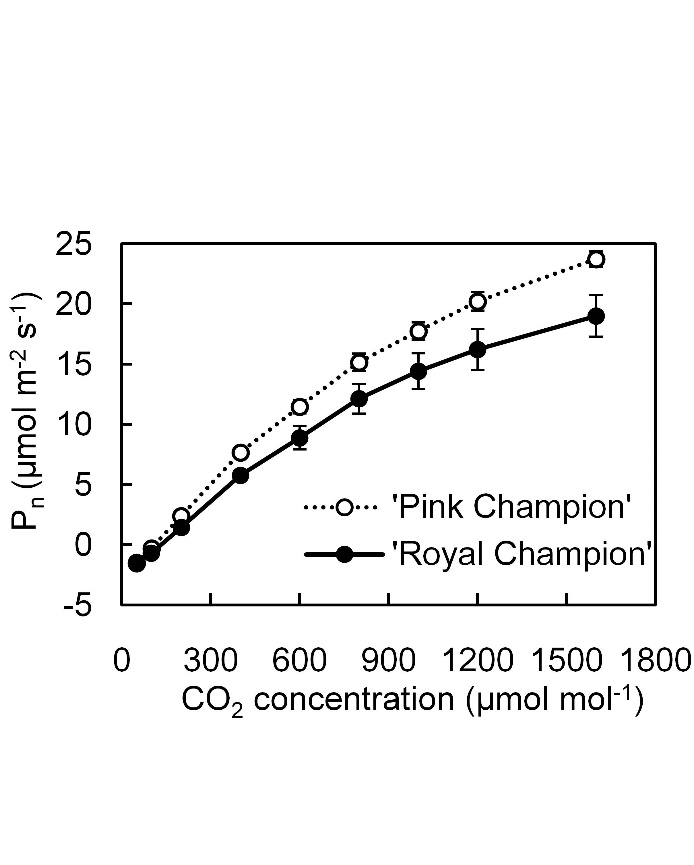


**Fig. S4.** CO_2_ response of net leaf photosynthesis (P_n_) of the fully expanded leaves in ‘Pink Champion’ and ‘Royal Champion’. This measurement was carried out with the portable gas exchange device equipped with a leaf chamber fluorometer (LI-6400XT; LI-COR, Lincoln, USA) at CO_2_ concentration between 50 and 1600 µmol mol^-1^. Six fully expanded leaves from six plants each were randomly selected for six response curves in each cultivar. The starting CO_2_ concentration was 400 µmol mol^-1^, followed by 200, 100, 50, 400, 600, 800, 1000, 1200, 1600 µmol mol^-1^; at each CO_2_ concentration, the measurements were taken after about 5 min. In the measurement chamber, photosynthetic photon flux density (PPFD, 10% blue, 90% red), leaf temperature and vapour pressure deficit (VPD) were maintained at 500 µmol m^-2^ s^-1^, 25 °C, and between 0.5-1 kPa, respectively. Vertical bars indicate standard error of mean (n=6).
